# Plasma lipids in Pseudoxanthoma Elasticum (PXE) patients: A comparative study with population-based reference values and Non-PXE controls

**DOI:** 10.1016/j.athplu.2023.12.003

**Published:** 2023-12-17

**Authors:** Iris M. Harmsen, Frank L.J. Visseren, Madeleine Kok, Pim A. de Jong, Wilko Spiering

**Affiliations:** a Department of Vascular Medicine, University Medical Centre Utrecht, Utrecht University, Utrecht, the Netherlands; b Department of Radiology, University Medical Centre Utrecht, Utrecht University, Utrecht, the Netherlands

## Abstract

**Background and aims:**

– Pseudoxanthoma elasticum (PXE) is a rare genetic disease caused by pathogenic mutations in the ABCC6 gene, resulting in low values of inorganic pyrophosphate (PPi). While low PPi is thought to contribute to arterial calcification, it remains unclear whether this fully explains premature calcification in PXE. It has been hypothesized that the ABCC6 gene could be related to dyslipidemia, which could contribute to vascular calcification seen in PXE. The aim of this study is to evaluate the relation between PXE and plasma lipid concentrations in a large cohort of PXE patients compared with reference values for the general population and compared with non-PXE controls.

**Methods:**

– The plasma concentrations of total cholesterol, HDL-cholesterol, tiglycerides, and LDL-cholesterol of 312 PXE patients were compared to age- and sex-matched modeled data of the general Dutch population. Differences in median lipid levels were compared with Mann-Whitney-U test. Secondly, plasma lipid concentrations of 44 PXE patients were compared to 44 not-genetically related relatives (spouses or friends), with linear models adjusted for age, sex and BMI.

**Results:**

– Total cholesterol in PXE patients was 5.6 [IQR 4.6–6.4] mmol/L versus 5.3 [IQR 4.7–6.0] mmol/L (p < 0.01) in the general population; triglycerides were 1.1 [IQR 0.9–1.7] mmol/L versus 1.0 [0.7–1.4] mmol/L (p < 0.01); HDL-c was 1.4 [IQR 1.2–1.7] mmol/L versus 1.5 [IQR 1.2–1.8] mmol/L (p = 0.03) and LDL-c was 3.3 [IQR 2.7–4.1] mmol/L versus 3.2 [IQR 2.7–3.8] mmol/L (p = 0.01). In the patient control analysis with 44 pairs and age, sex and BMI adjusted, comparison with the non-PXE controls only triglycerides were significantly different (mean difference: 0.38 (0.13–0.63)).

**Conclusion:**

–The lipid profiles of PXE patients are marginally different from the general population or compared to a matched control group, but the differences are unlikely to be clinically relevant**.** It is therefore unlikely that plasma lipids contribute to the premature vascular calcifications in PXE patients.

## Highlights

• PXE is a rare genetic calcification disease caused by ABCC6 gene mutations, resulting in low levels of PPi.
• PPi levels do not explain arterial calcification, so atherosclerosis due to ABCC6-related dyslipidemia could contribute.
• PXE patients exhibit no clinically relevant differences in levels of plasma lipids compared to the general population.
• This study concludes that plasma lipid levels are unlikely contributors to the premature arterial calcification in PXE patients.

## 1 Introduction

Pseudoxanthoma elasticum (PXE) is a rare autosomal recessive genetic disease with an estimated prevalence of 1 in 25,000–50,000 worldwide [1,2]. It is caused by pathogenic variants in the *ABCC6* gene, and is characterized by ectopic calcifications of elastin in the skin, Bruch's membrane in the retina and arterial wall [[3], [4], [5]], resulting in premature vascular disease [6,7].

According to the current hypothesis vascular calcification in PXE patients consist mainly of medial arterial calcification, due to a diminished amount of the calcification inhibitor inorganic pyrophosphate (PPi) [8,9]. The pathogenic variants in the *ABCC6* gene affect the ABCC6 transporter, which is thought to act as an ATP transporter in hepatocytes, resulting in a lower substrate for ENPP1, which converts ATP to ADP and PPi [10]. Studies have failed, however, to show a clear relationship between vascular calcification and PPi levels in PXE patients [[11], [12], [13]], suggesting that there are other processes contributing to the increased vascular calcification in PXE. Especially because the complete function of the ABCC6 transporter and its substrate is not yet elucidated [9,14,15].

*ABCC6* is part of the superfamily ATP cassette-binding (ABC) genes, that code for cell-membrane-transporters, of which many are related to lipid transport [16]. Certain *ABCC6* polymorphisms have been associated with lower plasma triglycerides and higher HDL-c levels in a non-PXE population [16]. Additionally, certain *ABCC6* haplotypes were more often found in a population of coronary heart disease patients (CHD), versus controls without CHD [17]. One of the additional pathways contributing to vascular calcification in PXE could therefore be atherosclerosis due to ABCC6-related dyslipidemia [18,19].

Previous research on plasma lipids *ABCC6 −/−* mice, a model for PXE, however, has shown conflicting results. There are three studies [[20], [21], [22]] reporting a decreased HDL-c and an increased triglyceride levels in *ABCC6* −/− mice compared to wild-types. Lower HDL-c levels in combination with increased triglyceride levels could indicate an influence of *ABCC6* on the reverse cholesterol pathway. An increase in LDL-c was observed in knockout mice, which is not consistent with this hypothesis. Fibroblasts of PXE patients have been shown to have increased expression of HMGcoA-reductase, increased levels of PCSK9 and reduced levels of APOE [23]. A small study in PXE patients (n = 32) compared with 14 non-PXE controls showed lower HDL-c (1.31 ± 0.06 mmol/L vs. 1.77 ± 0.13 mmol/L), total cholesterol, triglycerides and LDL-c were not significantly different between the groups [22]. These studies do not provide a clear picture of the effect of *ABCC6* on plasma lipids.

The aim of this study is to evaluate the relation between PXE and plasma lipid concentrations in a large cohort of PXE patients compared with reference values for the general population and compared with matched controls.

## 2 Methods

### 2.1 PXE population

The PXE patients were consecutive adult patients attending the Dutch Expertise Center for Pseudoxanthoma Elasticum (DECP), in the University Medical Center Utrecht, with a definitive or probable diagnosis of PXE according to the Plomp cirteria [4] (Supplementary Table 1). All subjects attending the DECP are asked to participate in the Dutch PXE Registry: a prospective cohort study using pseudonymized data from regular care visits. For this study, no additional interventions were performed on the patient group. Data on sex, age, body mass index (BMI) and lipid-lowering therapy (LLT) were collected for PXE patients at the first visit to the treating physician. For PXE patients, fasting plasma values of total cholesterol, triglycerides and HDL-c were obtained in the context of regular care, using enzymatic colorimetric methods (AU5811 analyzers, Beckman and Coulter). LDL-c was calculated using the Friedewald formula [24]. Diabetes was defined as the use of glucose-lowering drugs or a plasma glucose >7.0 mmol/L. Hypertension was defined as the use of antihypertensives or, when the average of three consecutive measurements showed a systolic blood pressure >140 mmHg, or a diastolic blood pressure >90 mmHg. Previous cardiovascular disease was defined as a history cardiovascular event.

If PXE patients were using LLT at the time of blood collection, the pre-treatment values of total cholesterol, triglycerides, HDL-c, and LDL-c were calculated, using data from meta-analyses on the lipid-lowering effects of these drugs. For every type and dose of LLT a different correction factor was applied to the lipid level after treatment (Supplementary Table 2) [[25], [26], [27]].

### 2.2 Population-based reference values for lipids

The distribution of total cholesterol, triglycerides, HDL-c, and LDL-c of our PXE population was compared to that of values in an age and sex matched simulated cohort. This cohort was based on publicly available reference values for the Dutch general population [28], which are previously identified through the use of the Lifelines cohort [29], a large population-based prospective cohort study in the Netherlands. The reference values are based on patients without lipid-lowering therapy. Total cholesterol and HDL-c are assessed using colorimetric assay. Triglycerides are measured using an assay based on glycerol phosphate oxidase-peroxidase. LDL-c was calculated with the Friedewald formula [28]. A broad range of percentiles for total cholesterol, triglycerides, HDL-c, and LDL-c are reported (1st, 2.5th, 5th, 10th, 25th, 50th, 75th, 90th, 95th, 97.5th and 99th), for men and women separately in age categories of 5 years. These reference values are considered as the standard in the Netherlands and are frequently used in an online tool (https://www.lipidtools.com/nl/) for identifying lipid disorders. With these extensively reported range of percentiles, it was possible to simulate a reference cohort. For every age- and sex group in which the percentiles are reported (e.g. men of 50–55 years), the best fitting distribution for each group was assessed for the given percentiles using the R-package “rriskDistributions” [30]. This supplied us with distribution parameters, e.g., mean and standard deviation, for every age- and sex-specific group. Total cholesterol, triglycerides, HDL-c, and LDL-c were found to follow a lognormal distribution in every group. With their distribution parameters, samples (n = 1000) were created for each group, forming a simulated reference cohort of 28,000 observations. Lastly, all PXE patients were categorized into the same age- and sex-specific groups, and for each patient, ten controls from the simulated reference cohort were randomly selected. This process resulted in a reference cohort with similar age and sex distribution to our PXE cohort (Supplementary Fig. 1).

### 2.3 PXE patient and control pairs

In addition to the simulated reference cohort, the plasma lipid levels in PXE patients were also compared to non-PXE controls, this allowed us to control for more potential confounders. The control population comprised of spouses and close friends of PXE patients, and are assumed to have a similar lifestyle to their friends and partners. The variables, including age, sex, BMI, medication use, and lipid levels, were collected in the context of a different patient-control study [11]. Lipids were analyzed in the same way as in PXE patients: Total cholesterol, HDL-c and triglycerides were analyzed using enzymatic colorimetric methods (AU5811 analyzers, Beckman and Coulter), LDL-c was calculated using the Friedewald formula. However, due to the difference in outcomes of interest of the patient-control study for which the data was collected, the control population was not fasted for blood collection. Pre-treatment levels of total cholesterol, triglycerides, HDL-c, and LDL-c were computed in the same manner as for the PXE patients. Only PXE patients who formed a pair with a close friend or spouse were included in this patient-control analysis to minimize any lifestyle-related influence (Supplementary Fig. 1).

### 2.4 Data analyses

Continuous variables with a normal distribution are depicted as mean ± standard deviation, while non-normally distributed continuous variables are presented as median (interquartile range (IQR)). Categorical variables are presented as number (%). Subjects with missing outcome variables (total cholesterol, triglycerides, HDL-c, and LDL-c), were excluded from the analysis (n = 11). There was one PXE patient with a missing BMI, which was imputed by single imputation using predictive mean matching.

The lipid values of the PXE cohort were compared with the simulated reference cohort (derived from the Dutch population references) with a Mann-Withney-U-test, because the distributions were not normally distributed. A sensitivity analysis was performed where only subjects without LLT were included.

In the patient control analysis (with close friends and spouses), population differences were assessed with T-test for normally distributed variable, Mann-Whithney *U* test for non-normally distributed variables, and Chi-square test for categorical variables. Furthermore, linear models were used to test the difference in lipid values between groups, both univariately and adjusted for potential confounders (age, sex and BMI). Triglycerides were log-transformed because of the non-gaussian distribution, and therefore a relative difference between patients and controls is displayed.

All analysis were performed with R version 4.2.2 (2022-10-31), using R-studio RStudio (2023-03-1).

### 2.5 Ethics

The study was approved by the Institutional Review Board of the UMC Utrecht (numbers: 16–622 and 18–767). Written informed consent was obtained from all participants for blood collection, and PXE patients provided written informed consent for the utilization of their medical files in research. The study protocols conform to the ethical guidelines of the 1975 Declaration of Helsinki.

## 3 Results

### 3.1 Patient characteristics of the PXE cohort

In December 2021 the DCEP included 323 patients with a confirmed diagnosis of PXE according to the Plomp criteria [4] and age 18 years or older (Supplemental Table 1). Eleven of them were excluded due to missing outcome measures (total cholesterol, triglycerides, HDL-c, and LDL-c), leaving a total of 312 patients (Supplementary Fig. 1). PXE patients were 52 ± 4 years old and 39 % was male and 54 patients (17 %) had a history of cardiovascular disease. The use of LLT was prevalent in this population with 98 patients taking a statin (29 %), and 15 patients taking ezetimibe (5 %). Total cholesterol was 5.1 ± 1.2 mmol/L, triglycerides were 1.1 [IQR 0.8–1.6] mmol/L, HDL-c in men was 1.3 ± 0.3 mmol/L, in women 1.6 ± 0.4 mmol/L, and LDL-c was 3.0 ± 1.0 mmol/L (Table 1).

**Table 1:** Characteristics of the PXE population.

n	312
Age (years)	52 (14)
Sex (male), n (%)	123 (39)

History of CVD, n (%)	
cardiac	13 (4)
Cerebrovascular	20 (6)
Peripheral	12 (4)
Multi	9 (3)
none	258 (83)

Smoking status, n (%)	
Current, daily smoker	45 (15)
Current, someday smoker	5 (2)
Former smoker	144 (47)
Never smoker	115 (37)

Diabetes, n (%)	12 (0)
Hypertension, n (%)	133 (43)
BMI (kg/m2)	26.4 (4.8)
eGFR (ml/min/1.73m2)	94 [82–103]

Total cholesterol (mmol/L)	5.1 (1.2)
Triglycerides (mmol/L)	1.1 [0.8–1.6]
HDL-c (mmol/L)	
Men	1.3 (0.3)
Women	1.6 (0.4)
LDL-c (mmol/L)	3.0 (1.0)

Statin, n (%)	
high intensity	16 (5)
moderate intensity	5 (2)
low intensity	71 (23)
None	220 (71)

Ezetimibe, n (%)	15 (5)

Data are reported as mean (SD), n (%) or median [IQR]. BMI = body mass index. eGFR = estimated glomerular filtration rate using Chronic Kidney Disease Epidemiology Collaboration (CKD-EPI) formula. Percentages are rounded to a whole number.

### 3.2 Lipid values in PXE patients compared with the general population

**Fig. 1:**
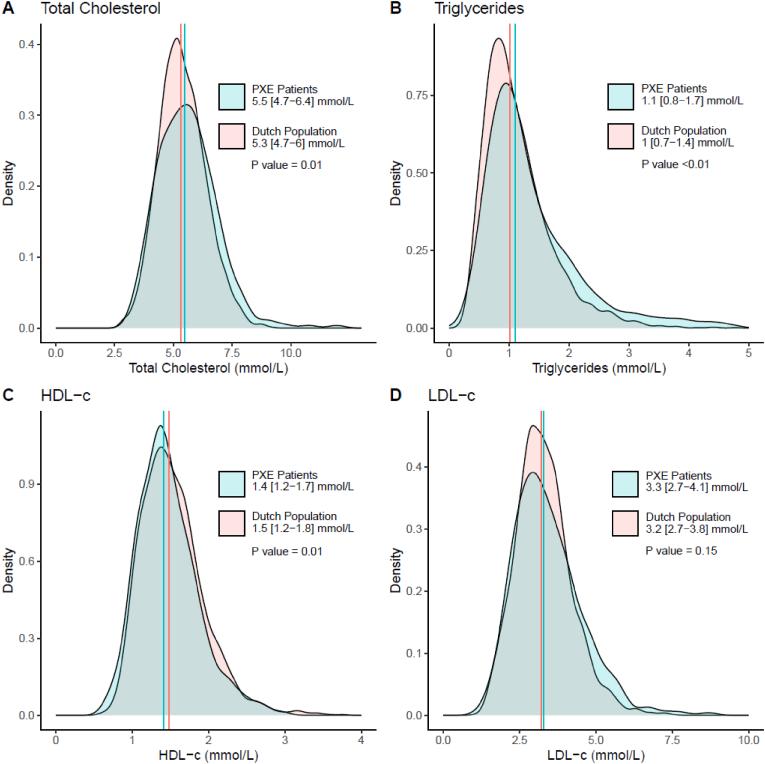
Distribution of plasma lipids in PXE Patients Distribution of plasma lipids in PXE Patients (n = 312) and simulated cohort based on Dutch Population Reference Values Distribution of total cholesterol (A), triglycerides (B), HDL-cholesterol (C) and LDL-cholesterol (D) after reverse calculation for lipid lowering therapy displayed in density plots for PXE Patients (blue) and the Dutch Population (red), the median is marked with a vertical line in the corresponding colours. P values for the difference between the groups are derived with Mann-Withney *U* test. Legend displays population median and [IQR]. (For interpretation of the references to color in this figure legend, the reader is referred to the Web version of this article.)

After recalculating the off-treatment lipid levels for PXE patients (n = 92) with lipid-lowering therapy, differences in plasma lipids concentrations of PXE patients compared with the simulated population cohort were small, yet statistically significant different for all the plasma lipids. The median [IQR] total cholesterol in PXE patients was 5.6 [IQR 4.6–6.4] mmol/L versus 5.3 [IQR 4.7–6] mmol/L in the general population (p < 0.01); triglycerides were 1.1 [IQR 0.9–1.7] mmol/L in PXE patients versus 1.0 [IQR 0.7–1.4] mmol/L in the general population (p < 0.01); HDL-c in PXE patients was 1.4 [IQR 1.2–1.7] mmol/L versus 1.5 [IQR 1.2–1.8] mmol/L (p = 0.03). LDL-c was 3.3 [IQR 2.7–4.1] mmol/L in PXE patients and was 3.2 [IQR 2.7–3.8] mmol/L in the general population (p = 0.01) (Fig. 1).

**Fig. 2:**
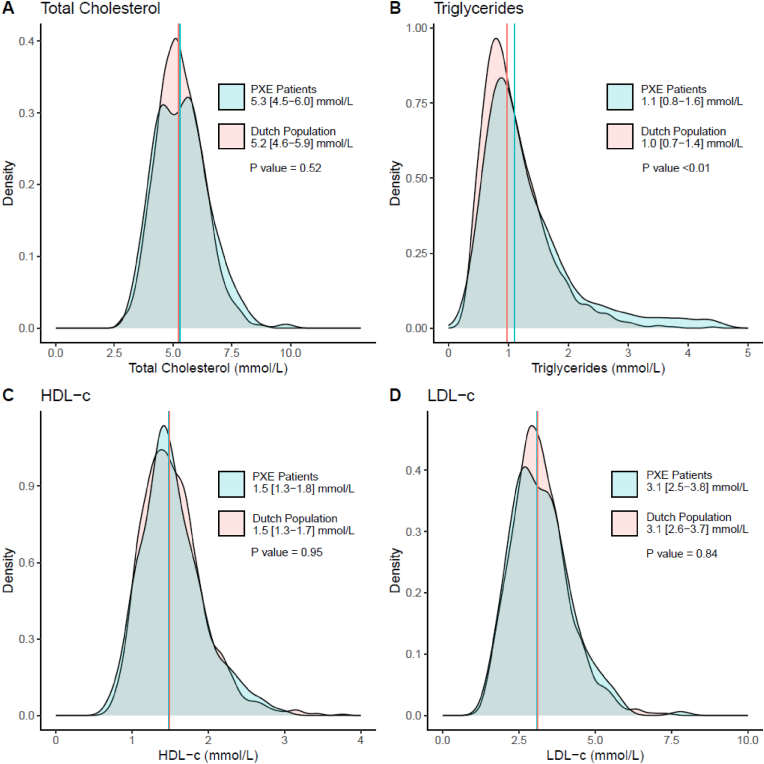
Distribution of plasma lipids in PXE Patients without statins Distribution of plasma lipids in PXE Patients without statins (n = 218) and simulated cohort based on Dutch Population Reference Values Distribution of total cholesterol (A), triglycerides (B), HDL-cholesterol (C) and LDL-cholesterol (D) displayed in density plots for PXE Patients (blue) and the Dutch Population (red), the median is marked with a vertical line in the corresponding colours. P values for the difference between the groups are derived with Mann-Withney *U* test. Legend displays population median and [IQR]. (For interpretation of the references to color in this figure legend, the reader is referred to the Web version of this article.)

Additionally, PXE patients not using lipid-lowering therapy at the time of sampling (n = 218) were compared with the simulated population cohort with similar age and sex distribution, in a sensitivity analysis. This analysis revealed an attenuation of the differences in total cholesterol, HDL-c, and LDL-c (Fig. 2). Only triglycerides remained slightly, but statistically significantly higher in PXE patients compared to the general population (1.1 [IQR 0.8–1.6] mmol/L vs 1.0 [IQR 0.7–1.4] mmol/L) (Fig. 2).

### 3.3 Characteristics and differences in plasma lipid concentrations of PXE patient and control pairs

**Fig. 3:**
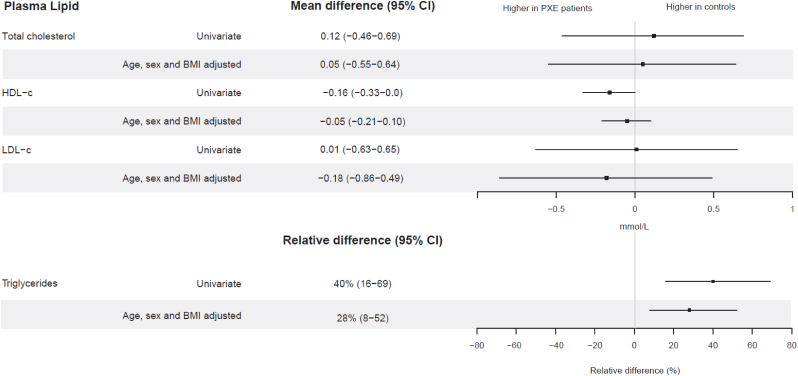
Forest plot of the difference in plasma lipids between PXE patients and controls Mean difference and (95 % CI) of total cholesterol, HDL-c, and LDL-c between PXE patients and controls estimated using linear models, univariate and adjusted for age, sex, and BMI. Relative difference and (95 % CI) of triglycerides are derived from linear models: univariate and adjusted for age, sex, and BMI. The percentage difference is calculated by exponentiating the estimates derived from the model, subtracting one, and multiplying by 100.

There were 44 pairs of a PXE patient with a control. Patients and controls were similar in age and BMI: 58 ± 10 year versus 61 ± 10 years and 26.0 ± 4.3 kg/m^2^ versus 25.7 ± 3.7 kg/m^2^, respectively. Controls were more often male (64 %) compared to the PXE patients (30 %). Statin use was higher in PXE patients (50 %) than in controls (18 %), as was ezetimibe use (11 % versus 2 %) (Table 2). After adjustment for confounding factors (age, sex, and BMI) only triglycerides were found to be significantly higher in controls versus PXE patients: 28 % (95 % CI 8–52) (Fig. 3). There was no statistically significant difference in total cholesterol, HDL-c, and LDL-c between PXE patients and controls. After adjustment for confounding the mean differences were 0.05 (95 % CI: −0.55–0.64) mmol/L; 0.04 (95 % CI: −0.2–0.12) mmol/L and 0.39 (95 % CI: −0.11–0.9) mmol/L, respectively (Fig. 3).

**Table 2:** Characteristics of Patient and control pairs.

	PXE patients	Controls	P value
n	44	44	
Age (years)	58 (10)	61 (10)	0.15
Sex (male), n (%)	13 (30)	28 (64)	<0.01
BMI (kg/m^2^)	26.0 (4.3)	25.7 (3.7)	0.72

Relationship with PXE patient			
Spouse, n (%)		42 (95)	
Friend, n (%)		2 (5)	

Total cholesterol (mmol/L)	5.1 (1.2)	5.9 (1.1)	<0.01
Triglycerides (mmol/L)	1 [0.7–1.4]	1.6 [1.1–2]	<0.01
HDL-c (mmol/L)	1.7 (0.4)	1.5 (0.3)	
Men	1.3 (0.3)	1.4 (0.3)	0.73
Women	1.8 (0.4)	1.6 (0.3)	0.10
LDL-c (mmol/L)	2.9 (1.0)	3.6 (1.0)	0.002

Statin, n (%)			<0.01
high intensity	4 (9)	0 (0)	
moderate intensity	1 (2)	0 (0)	
low intensity	17 (39)	8 (18)	
none	22 (50)	36 (82)	

Ezetimibe, n (%)	5 (22)	1 (2)	0.22

Off-treatment lipid levels			
Total cholesterol (mmol/L)	6.1 (1.5)	6.2 (1.2)	0.69
Triglycerides (mmol/L)	1.1 [0.9–1.5]	1.6 [1.2–2.1]	<0.01
HDL-c (mmol/L)	1.6 (0.5)	1.5 (0.3)	
Men	1.3 (0.3)	1.4 (0.3)	0.31
Women	1.8 (0.4)	1.6 (0.4)	0.20
LDL-c (mmol/L)	3.9 (1.9)	4.0 (1)	0.97

Characteristics of the matched subgroup of patients and controls Data are reported as mean (SD), n (%) or median [IQR]. BMI = body mass index. eGFR = estimated glomerular filtration rate using Chronic Kidney Disease Epidemiology Collaboration (CKD-EPI) formula. Percentages are rounded to a whole number.

## 4 Discussion

The present study demonstrates that plasma lipid concentrations in PXE patients are only marginally different compared to the general population reference values. Total cholesterol, HDL-c and LDL-c levels are not significantly different from those of healthy controls. These findings imply that the lipid profiles of PXE patients are not more atherogenic than those of the general population. The small differences in plasma lipids are unlikely to contribute to premature vascular disease in PXE patients. Therefore, our study indicates that dyslipidemia is not a primary manifestation of PXE.

In a previous study in PXE patients the HDL-c plasma concentration was found to be lower compared to healthy controls, which contrasts the present study [22]. However, it should be noted that both the group of PXE patients and the control group in that study were relatively small, with only n = 32 and n = 14 participants respectively. Additionally, there is limited information presented on both groups with regards to the origin population and the comparability of both groups. It is therefore challenging to accurately assess and identify the differences between our studies. Lastly, lifestyle and BMI were not accounted for in their analysis.

Previous studies on ABCC6 gene mutations in animal models and in vitro have produced conflicting results regarding their impact on lipid levels. Some studies suggest lower HDL-c and higher triglycerides, while others indicate increased LDL-c and altered gene expressions related to cholesterol formation [[20], [21], [22], [23]]. Thus, the findings of previously reported studies do not support a single biological plausible effect of *ABCC6* mutations on the lipid spectrum. We can therefore only conclude that currently there is no definitive evidence linking PXE with any specific pattern of dyslipidemia. The findings of our current study also reinforce this lack of association. This, and especially the absence of evidence for increased LDL-c in PXE, makes the possible alternative pathway of vascular calcification through increased plaque formation, or atherosclerosis, rather than PPi related arterial calcification less likely.

Although we find no evidence for a more atherogenic lipid profile in PXE patients, lipid-lowering therapy could still be important for this group. Because of the arterial calcification caused by PXE, they are considered to be at a higher risk of cardiovascular events, and statin treatment is one of the cornerstones of cardiovascular risk management (CVRM) [31]. Because of this, we see a relatively high proportion of lipid-lowering treatment (29 %) in our PXE population, which is why we have opted to calculate pre-treatment levels. Due to the rarity of PXE, there are no specific guidelines for this group, the physician and the patient should make CVRM decisions together.

### 4.1 Strengths and limitations

The major strength of this study is the substantial number of PXE patients included and the systematic documentation of lipid levels in the adult PXE population. Additionally, we calculated the pre-treatment levels of all the patients in our population that already used lipid-lowering treatment.

A limitation to this study is that we did not have patient-level data from a general population cohort and were therefore not able to adjust for possible confounding covariates, or able to perform subgroup analysis. With the current approach, however, we were able to correct for age and sex differences, which are considered to be the most important confounding variables.

The group of controls, family and friends of PXE patients, is small. This impacts the power of the comparison between the groups. Another limitation is that the 44 matched controls were not similarly instructed with regards to the blood collection. The significantly higher triglycerides we found in the control group could be since they were not instructed to be fasted for their blood collection.

Lastly, although calculating pre-treatment levels of lipid values is common practice, it is not perfect. The factors that were used for calculation are based on data from clinical trials. It is common knowledge that people that participate in trials are more compliant than the general population. This could mean that the lipid values for the PXE patients are slightly overestimated and is probably the reason why the differences between the PXE patients and Dutch reference groups are attenuated when excluding PXE patients with LLT.

## 5 Conclusion

The lipid profiles of PXE patients are at most marginally different from the general population or compared to a matched control group, but the differences are unlikely to be clinically relevant. It is therefore unlikely that plasma lipids contribute to the typical premature vascular calcification in PXE patients.

## Declaration of competing interest

The authors declare that they have no known competing financial interests or personal relationships that could have appeared to influence the work reported in this paper.
