# Supplementary material for: Plasma lipids in Pseudoxanthoma Elasticum (PXE) patients: A comparative study with population-based reference values and Non-PXE controls
Source: Atheroscler Plus. 2023 Dec 17;55:5–11. doi: 10.1016/j.athplu.2023.12.003 (PMC10784135; doi:10.1016/j.athplu.2023.12.003)
Supplement: Multimedia component 1 [file mmc1.docx]

# Supplementary Material

Content:

1. Supplementary table 1 – Plomp criteria used for the diagnosis of PXE
2. Supplementary table 2 – Correction factors used for Lipid-lowering Therapy.
3. Supplementary table 3 - Overview of statin and ezetimibe use.
4. Supplementary Figure 1 – Flow chart

## **Supplementary Table 1** – Plomp criteria used for the diagnosis of PXE.

Criteria are adapted from Plomp et al.^4^

| Major Diagnostic Criteria | |
| --- | --- |
| Skin | a. Yellowish papules and/or plaques on the lateral side of the neck and/or flexural areas of the body; *or* |
|  | b. Increase of morphologically altered elastin with fragmentation, clumping and calcification of elastic fibers in a skin biopsy taken from clinically affected skin |
| Eye | a. Peau d'orange of the retina; *or* |
|  | b. One or more angioid streaks, each at least as long as one disk diameter. When in doubt, fluorescein or indocyanine green angiography of the fundus is needed for confirmation. |
| Genetics | a. A pathogenic mutation of both alleles of the *ABCC6* gene; *or* |
|  | b. A first-degree relative (parent, sib, child) who meets independently the diagnostic criteria for definitive PXE |

| Minor Diagnostic Criteria | |
| --- | --- |
| Eye | a. One angiod streak shorter than one disk diameter; *or* b. One or more ‘comets’ in the retina; *or* c. One or more ‘wing signs’ in the retina |
| Genetics | a. A pathogenic mutation of one allele of the *ABCC6* gene |

| Requirements for the diagnosis of PXE | |
| --- | --- |
| Definitive Diagnosis | The presence of two (or more) major criteria not belonging to the same (skin, eye, genetic) category. |
| Probable Diagnosis | The presence of two major eye or two major skin criteria, *or* |
|  | The presence of one major criterion and one or more minor criteria not belonging to the same category as the major criterion |
| Possible Diagnosis | The presence of a single major criterion, *or* |
|  | The presence of one or more minor criteria |

## **Supplementary Table 2** - Correction factors used for Lipid-lowering Therapy

Correction of Total Cholesterol

|  | Dosis in mg/day | Correction factor |
| --- | --- | --- |
| Atorvastatin | 10 | 1.36 |
|  | 20 | 1.44 |
|  | 40 | 1.51 |
|  | 80 | 1.61 |
| Fluvastatin | 20 | 1.17 |
|  | 80 | 1.33 |
| Pravastatin | 40 | 1.26 |
|  | 20 | 1.25 |
| Rosuvastatin | 5 | 1.41 |
|  | 10 | 1.49 |
|  | 40 | 1.68 |
| Simvastatin | 10 | 1.27 |
|  | 20 | 1.27 |
|  | 40 | 1.35 |
| Ezetimibe | 10 | 1.11 |

Correction of triglycerides

|  | Correction factor |
| --- | --- |
| Atorvastatin | 1.2 |
| Fluvastatin | 1.11 |
| Pravastatin | 1.14 |
| Rosuvastatin 5 mg | 1.18 |
| Rosuvastatin >5 mg | 1.21 |
| Simvastatin | 1.08 |
| Ezetimibe | 1.13 |

Correction of HDL-c

|  | Correction factor |
| --- | --- |
| Atorvastatin | 0.93 |
| Fluvastatin | 0.93 |
| Pravastatin | 0.89 |
| Rosuvastatin | 0.91 |
| Simvastatin | 0.94 |
| Ezetimibe | 0.93 |

Correction of LDL-c

|  | Dosis in mg/ day | Correctionfactor |
| --- | --- | --- |
| Atorvastatin | 10 | 1.59 |
|  | 20 | 1.75 |
|  | 40 | 1.96 |
|  | 80 | 2.22 |
| Fluvastatin | 20 | 1.27 |
|  | 80 | 1.49 |
| Pravastatin | 40 | 1.64 |
|  | 20 | 1.32 |
| Rosuvastatin | 5 | 1.61 |
|  | 10 | 1.75 |
|  | 40 | 2.13 |
| Simvastatin | 10 | 1.37 |
|  | 20 | 1.47 |
|  | 40 | 1.59 |
| Ezetimibe | 5/ 10 | 1.35 |

## **Supplementary Table 3** - Overview of statin and ezetimibe use.

| **LLT** | **Number of patients** |
| --- | --- |
| atorvastatin 10 mg | 7 |
| atorvastatin 20 mg | 7 |
| atorvastatin 40 mg | 14 |
| atorvastatin 80 mg | 2 |
| fluvastatin 20 mg | 1 |
| fluvastatin 80 mg | 1 |
| pravastatin 20 mg | 2 |
| pravastatin 40 mg | 4 |
| rosuvastatin 10 mg | 7 |
| rosuvastatin 5 mg | 11 |
| simvastatin 10 mg | 2 |
| simvastatin 20 mg | 12 |
| simvastatin 40 mg | 22 |
| Ezetimibe | 15 |

##
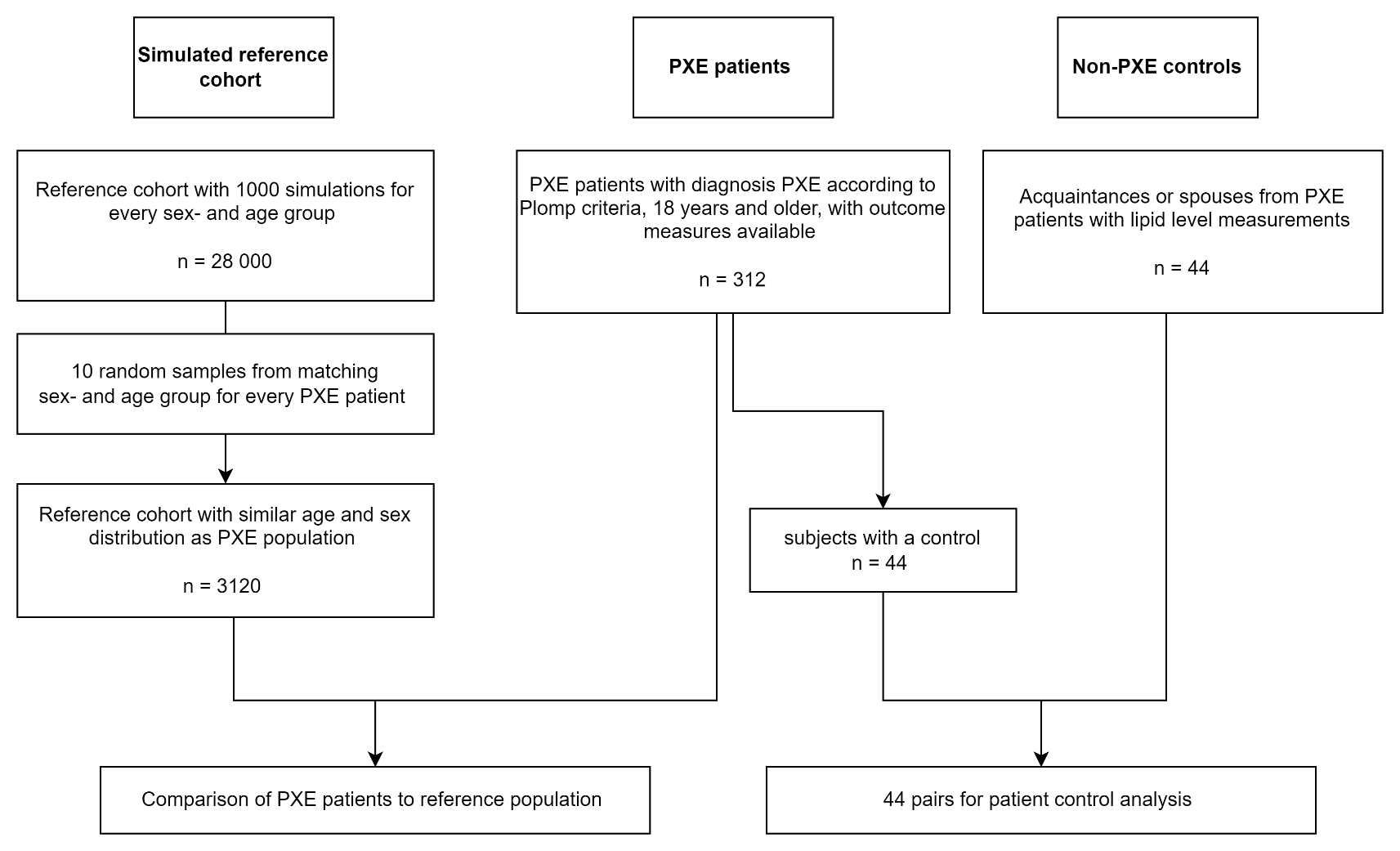
**Supplementary Figure 1**

Flow-chart depicting the simulated reference cohort, PXE patients and non-PXE controls assessed in this study.
